# Parenting on a digital leash: time pressure, parenting guilt, and emotional exhaustion in Chinese dual-earner families

**DOI:** 10.3389/fpsyg.2026.1774016

**Published:** 2026-07-24

**Authors:** Zhongya Ji, Yao Lu, Guanxin Yao

**Affiliations:** Yangzhou University, Yangzhou, China

**Keywords:** digitally-mediated parenting, emotional exhaustion, time pressure, parenting guilt, dual-earner families

## Abstract

**Background:**

Digital tools are increasingly integrated into parenting, but their psychological costs remain unclear. This study investigates whether digitally-mediated parenting intensity predicts emotional exhaustion among Chinese dual-earner parents, and whether time pressure and parenting guilt mediate this relationship.

**Methods:**

A cross-sectional survey was conducted with 713 dual-earner parents from 45 communities across 13 cities in Jiangsu Province, China. Participants completed measures of digital parenting intensity, time pressure, parenting guilt, and emotional exhaustion. We tested the measurement model and the hypothesized mediation paths with item-level latent-variable structural equation modeling (SEM), using maximum likelihood estimation and 5,000 bootstrap samples.

**Results:**

The four-factor measurement model fitted the data well, χ^2^(59) = 92.13, *p* = 0.004, CFI = 0.990, TLI = 0.987, RMSEA = 0.028, SRMR = 0.026, and fitted substantially better than a single-factor model. In the structural model, digital parenting intensity predicted emotional exhaustion directly [β = 0.294, 95% CI (0.184, 0.422)] and indirectly through time pressure [β = 0.099, 95% CI (0.016, 0.184)] and parenting guilt [β = 0.157, 95% CI (0.090, 0.228)]. The total indirect effect was also significant [β = 0.256, 95% CI (0.180, 0.340)].

**Conclusion:**

Digitally mediated parenting was associated with emotional exhaustion through time pressure and parenting guilt. In this study, the “digital leash” was reflected in school communication, online check-ins, parenting information seeking, and public sharing, which kept dual-earner parents connected to childcare demands even outside the family setting.

## 1 Introduction

The pervasive integration of digital technologies into the family sphere has fundamentally reconfigured contemporary parenting globally (Choy et al., 2024; Türen and Kahraman, 2025). In recent China, a suite of tools—from parenting apps and online homework platforms to the constant connectivity of messaging groups—promises to enhance oversight, streamline caregiving, and foster family connection. However, the manifestation and consequences of this digital intensification are profoundly shaped by local socioeconomic structures and cultural norms (Chang et al., 2024). The proliferation of Internet-based platforms has given rise to a distinctive socio-cultural phenomenon in China known as “shai,” a term derived from the English word “share.” This practice encapsulates the innate human desire for communication and socialization, which has been dramatically amplified by digital technologies that significantly lower the cost of sharing (Choy et al., 2024; Türen and Kahraman, 2025). As a cornerstone of this digital ecosystem, WeChat has become deeply embedded in the daily life of Chinese society. Tencent's 2018 report highlighted the platform's immense scale, with over 1.082 billion monthly active users and 45 billion daily messages sent, underscoring its role as a fundamental lifestyle infrastructure (Pang and Ruan, 2023). Within this context, Chinese netizens actively “shai” their personal lives on WeChat Moments and group chats, instantly publicizing a spectrum of experiences and emotions—from joy and happiness to frustration and sorrow. The “sharing” of parenthood, particularly among urban young parents, represents a prominent manifestation of this trend. While communication scholars often categorize sharing into four modes—personal, collaborative, public, and civic (Gore-Gorszewska, 2024)—the parenting practices observed on WeChat synthetically blend these forms. Centered predominantly on child-rearing, these once-private parenting experiences and emotions are now thrust into the public arena of social media (Wu W. F. et al., 2024). In the name of love, the boundary between the private family sphere and the public digital space becomes porous. Through the continuous sharing of parenting knowledge, behaviors, and sentiments, urban parents are collectively forging a novel virtual public space. This space, though intangible, is actively reshaping social norms and family ethics. Here, the phrase “digital leash” is used to describe how digital platforms keep parents connected to parenting demands across settings. School WeChat groups, online check-in tasks, parenting apps, and social media sharing can make childcare responsibilities follow parents into work hours and rest time. This does not mean that digital tools are only harmful; rather, it highlights the conditions under which convenience becomes another source of obligation.

A thematic analysis of the content shared by these parents reveals that “parent-child companionship” has emerged as a dominant and recurrent motif (Zhao et al., 2015). Expressions such as “companionship is the best education” permeate WeChat Moments, creating a dense, normative discourse around dedicated parenting time. This thematic focus is further reinforced and institutionalized through “check-in” tasks assigned by schools and kindergartens. Parents are frequently required to document activities—from reading books to completing physical exercises—and share visual evidence (photos or videos) in class groups or their personal networks. Consequently, the intimate act of parenting is increasingly transformed into a performative and socially monitored practice, illustrating a profound integration of digital sharing into the fabric of modern family life in China (Jin and Dai, 2025).

In many Western contexts, the dual-earner family model often operates within a framework of comparatively robust public childcare support and a cultural emphasis on individual nuclear family autonomy (Waismel-Manor and Levanon, 2024). In contrast, the Chinese dual-earner family model, particularly within the vast state-sector system (encompassing civil servants, and employees in state-owned enterprises, education, and healthcare), presents a distinct reality. These couples benefit from high job security but face unique pressures: rigid working hours, a culture of presenteeism, and income levels that often make full-time, dual careers a necessity rather than a choice (Fu et al., 2025). Consequently, these families heavily rely on intensive intergenerational support from grandparents for both childcare and financial co-residence, creating a tightly interdependent, multi-generational household system. It is within this specific, high-pressure context that digital parenting tools are adopted, not as simple conveniences, but as essential infrastructure for managing competing demands across work and complex family networks (Li et al., 2020).

A distinct and intensive parenting model has emerged in contemporary China, deeply intertwined with the nation's rapid digitalization and unique socioeconomic fabric. This model is characterized by what scholars term the “new ethics of accompaniment” (Zhang, 2024), where constant, attentive presence is not merely an ideal but a societal imperative for being a “good parent.” This norm is powerfully reinforced through digital mediation. Platforms like WeChat transform into virtual arenas where teachers, extended family, and peers exert a form of “square panopticon” surveillance—a continuous, low-intensity monitoring that mandates real-time responses to school notifications and the public performance of “parenting-by-posting” (e.g., sharing proof of completed parents-child activities; Vallejos, 2014). This digitally-facilitated oversight intensifies the inherent pressures within China's dual-earner family system, particularly for state-sector employees who benefit from job security but face rigid schedules and a culture of presenteeism. With limited access to affordable, high-quality external childcare, these families heavily rely on multi-generational co-residence, creating complex interdependencies (Hao et al., 2025). Beneath the veneer of efficiency, these technologies introduce novel and significant psychological demands. The constant stream of notifications from school groups, the pressure to perform “ideal parenting” on social media, and the obligation to complete digital tasks assigned by institutions transform parenting into an “always-on,” (Hao et al., 2025) high-pressure enterprise. This digitally-driven intensification consumes scarce time resources and fuels feelings of inadequacy against pervasive societal expectations, potentially leading to profound emotional exhaustion—a core dimension of parental burnout (Zhao et al., 2024; Schittek et al., 2025; Shnitzer-Meirovich et al., 2024). Within this high-stakes context, where educational achievement is paramount, digital tools become essential yet double-edged instruments (Lin et al., 2024; Yang et al., 2024). They are indispensable for managing logistical demands across work and complex kinship networks, but they also blur boundaries and amplify anxieties, embedding parents in a cycle of social comparison and guilt (Lamprianidou et al., 2025). Therefore, examining digitally-mediated parenting in China requires understanding it not as a simple convenience, but as a critical site where societal expectations, familial obligations, and technological pressures converge to shape parental wellbeing.

Existing research, largely rooted in Western settings, has traditionally focused on time-based and strain-based conflicts within nuclear families (Asiimwe et al., 2025; Pruett et al., 2024). A critical gap exists in empirically testing how the intensity of digitally-mediated parenting translates into emotional outcomes for parents within the unique Chinese dual-earner family's intergenerational support system. Although qualitative insights vividly portray the associated anxieties, large-scale quantitative evidence is lacking (Chen et al., 2021; Lu et al., 2016). We do not know whether the emotional cost arises primarily from the objective time pressure created by these technologies, the subjective parental guilt they engender, or a combination of both pathways. This study is situated within this research gap. Drawing on a unique survey of 713 parents from 45 local communities across 13 cities in Jiangsu, we propose and quantitatively test a parallel mediation model to explain the psychological mechanism linking digital parenting to emotional exhaustion in a representative Chinese regional context. We focus our investigation on Jiangsu Province, a microcosm of China's own socioeconomic spectrum. Its three distinct regions—the highly affluent and urbanized South (e.g., Suzhou, Wuxi), the developing North (e.g., Xuzhou, Lianyungang), and the transitional Central area—mirror the core-periphery dynamics found across many economies. Studying Jiangsu allows us to capture the experiences of parents at the forefront of technological adoption and economic pressure (the South), as well as those navigating different developmental realities, thereby enhancing the generalizability of our findings.

This study investigates two central research questions:

1. To what extent does the intensity of digitally-mediated parenting directly contribute to parental emotional exhaustion within the Chinese dual-earner context?
2. Do time pressure and parenting guilt serve as parallel psychological mechanisms that independently mediate this relationship?

This study shifts the analytical focus from how digital tools reshape parenting practices to whether and through what psychological mechanisms the intensity of digitally-mediated parenting leads to parental emotional exhaustion. To build a theoretical foundation for our quantitative model, this chapter integrates literature from two primary domains: the work-family interface and the psychology of parenting stress (Saunders et al., 2020; Zhao and Ju, 2022). We first establish the direct link between digitally-mediated parenting and emotional exhaustion, then theorize the parallel mediating roles of time pressure and parenting guilt, finally contextualizing this model within the unique pressures of China's dual-earner families.

Parental burnout, with emotional exhaustion as its core dimension, is a state of overwhelming exhaustion resulting from chronic parenting stress (Ren et al., 2024). The Conservation of Resources (COR) theory provides a robust framework for understanding this process (Aktu, 2024; Guo et al., 2024). COR theory posits that individuals strive to obtain, retain, and protect their valued resources, and psychological stress occurs when these resources are threatened or lost (Kim and Kerr, 2024; Mikolajczak et al., 2023). We conceptualize digitally-mediated parenting intensity—the pervasive demand to manage child-rearing through apps, mandatory online tasks, and performative social sharing (Lu et al., 2024; Wang et al., 2024)—as a significant chronic stressor that depletes parental resources. The constant cognitive switching between work and family domains prompted by digital notifications, the effort required to manage online parenting tasks, and the vigilance needed to curate a “good parent” identity online consume mental and emotional energy (Urbanowicz et al., 2024; Wang et al., 2025). This persistent resource drain, without adequate opportunity for recovery, is theorized to have a direct positive effect on emotional exhaustion (Ensanimehr et al., 2024).

Drawing on studies of work-family conflict, intensive parenting, Chinese dual-earner family arrangements, and the ethics of companionship (Li et al., 2020; Waismel-Manor and Levanon, 2024; Zhang, 2024; Zhao and Ju, 2022; Chuang et al., 2024), we use the term “dual ethical squeeze” to describe the combined pressure of two expectations: sustained work commitment and intensive parental companionship. This term is introduced in this article as a conceptual label rather than as an established framework. It helps organize our interpretation of the mediating pathways. Therefore, this study proposes and empirically tests a conceptual model that delineates the psychological pathways from digitally-mediated parenting intensity to parental emotional exhaustion. We posit a direct effect and further theorize two parallel mediating mechanisms–time pressure and parenting guilt–that independently transmit the influence of digital parenting intensity on exhaustion. The specific hypotheses are as follows:

***H1:*** *Direct effect*. We hypothesize that digitally-mediated parenting intensity exerts a significant positive direct effect on parental emotional exhaustion. The constant demands and cognitive load associated with pervasive digital childcare tasks are theorized to deplete psychological resources directly, leading to heightened exhaustion.

***H2:*** *First mediating pathway (time pressure)*. We hypothesize that time pressure serves as a critical mediating mechanism. Specifically, we propose that higher levels of digitally-mediated parenting intensity increase feelings of chronic time pressure (Path a1), which in turn, contributes to greater emotional exhaustion (Path b1). The statistical product of paths a1 and b1 is expected to be significant, indicating an indirect effect. This pathway captures the resource depletionprocess, where digital tools intensify the perceived scarcity of time, a fundamental personal resource.

***H3:*** *Second mediating pathway (Parenting Guilt)*. We hypothesize that parenting guilt serves as a distinct, parallel mediating mechanism. We propose that higher levels of digitally-mediated parenting intensity exacerbate feelings of parenting guilt (Path a2), which in turn, lead to greater emotional exhaustion (Path b2). The statistical product of paths a2 and b2 is also expected to be significant. This pathway captures the identity-based strainprocess, where digital platforms foster unrealistic parenting ideals and social comparisons, triggering guilt when these ideals are not met.

## 2 Method

The study was conducted in accordance with the Declaration of Helsinki. The survey was anonymous, involved adult participants, collected no identifiable personal information, and posed no more than minimal risk. Formal ethical approval was therefore waived in accordance with the institutional requirements applicable to this type of survey research. All participants were informed about the purpose of the study, the voluntary nature of participation, their right to withdraw, and the confidentiality of their responses. Electronic informed consent was obtained before participants completed the questionnaire.

A quantitative, cross-sectional survey was administered to dual-earner parents in Jiangsu Province, China. The target population was parents with at least one child aged 0–12 years, where both parents were engaged in full-time employment.

We adopted a multi-stage stratified sampling strategy to ensure broad geographical coverage across Jiangsu's diverse regional economies. The 13 prefecture-level cities in Jiangsu were classified into three strata: Southern Jiangsu (e.g., Suzhou, Wuxi, and Nanjing), Central Jiangsu (e.g., Yangzhou, Taizhou, and Nantong), and Northern Jiangsu (e.g., Xuzhou, Suqian, and Yancheng). To ensure a diverse sample, participants were recruited through a combination of online channels, including professional survey platforms and targeted posts on community and parenting forums, as well as offline channels, such as collaborations with kindergartens and community centers. A screening question at the beginning of the survey ensured respondents met the dual-earner and parental status criteria. The survey yielded 713 valid responses for analysis. Data was collected within 6 months *via* an online questionnaire. The sample demonstrated a reasonable distribution across the 13 target cities. Among the 713 valid responses, 62.4% of participants reported working in state-owned enterprises, public institutions (e.g., schools, hospitals), or government agencies. The remaining 37.6% worked in private or foreign-owned companies. Thus, while the sample includes a substantial proportion of state-sector employees, it also covers non-state-sector dual-earner parents. However, readers should be cautious when generalizing the findings to families in the informal economy or those without stable employment. Before testing the hypotheses, a comprehensive descriptive analysis of the sample demographics and core variables was conducted.

The data analysis proceeded in three stages. First, the dataset was screened for missing values, out-of-range responses, and unengaged response patterns; all 713 valid responses were retained. Descriptive statistics, Cronbach's alpha coefficients, Pearson correlations, and independent-samples *t* tests were then computed for the preliminary analyses. Common method bias was assessed using Harman's single-factor test. To analyse the four constructs at the item level, we conducted confirmatory factor analysis (CFA) and latent-variable structural equation modeling (SEM) using maximum likelihood estimation. The measurement model specified four correlated latent constructs: Digital Parenting Intensity (4 items), Time Pressure (3 items), Parenting Guilt (3 items), and Emotional Exhaustion (3 items). Model fit was evaluated using χ^2^, CFI, TLI, RMSEA, and SRMR. Convergent validity was evaluated using standardized factor loadings, composite reliability (CR), and average variance extracted (AVE).

In the structural model, Digital Parenting Intensity predicted Time Pressure, Parenting Guilt, and Emotional Exhaustion. Time Pressure and Parenting Guilt were modeled as two parallel mediators. Their residuals were allowed to correlate because the two variables capture related forms of parenting strain. Indirect effects were tested using 5,000 nonparametric bootstrap samples and 95% confidence intervals. An indirect effect was considered statistically significant when its 95% bootstrap confidence interval did not include zero.

## 3 Results

### 3.1 Descriptive statistics

Descriptive statistics were calculated for all 13 items across the four constructs. The means ranged from 3.09 to 3.37 on a 5-point Likert scale, and standard deviations ranged from 1.24 to 1.30, indicating moderate to high levels of agreement and variability in responses. The skewness and kurtosis values for all items were within acceptable ranges (between −1 and +1), suggesting that the data approximates a normal distribution (Table 1).

**Table 1:** Descriptive statistics for survey items and constructs (*N* = 713).

Construct	Item	Min	Max	Mean	Std. Deviation	Skewness	Kurtosis
Digital parenting intensity			3.185	1.022			
1.	Coping with school-assigned “check-in” tasks *via* WeChat consumes a lot of my energy.	1	5	3.216	1.275	−0.250	−0.995
2.	I often post about my child's daily life or achievements on platforms like WeChat Moments or Xiaohongshu.	1	5	3.180	1.280	−0.201	−1.038
3.	I spend a significant amount of time searching for and learning parenting knowledge on WeChat groups and public accounts.	1	5	3.198	1.273	−0.239	−0.980
4.	Overall, various parenting tasks on my smartphone make me feel burdened.	1	5	3.147	1.267	−0.191	−1.006
Time pressure			3.114	1.073			
5.	I always feel that time is not enough and I am rushing every day.	1	5	3.123	1.285	−0.155	−1.089
6.	I often need to handle work while simultaneously responding to family or my child's demands.	1	5	3.123	1.298	−0.156	−1.068
7.	Heavy parenting and work duties leave me no time for my own interests.	1	5	3.094	1.266	−0.139	−1.055
Parenting guilt			3.250	1.050			
8.	I feel guilty when I am unable to accompany my child due to a busy work schedule.	1	5	3.247	1.272	−0.330	−0.976
9.	I feel very self-blame after losing patience with my child.	1	5	3.236	1.240	−0.281	−0.922
10.	I often worry that I might hinder my child's development because I am not doing well enough.	1	5	3.268	1.254	−0.280	−0.932
Emotional exhaustion			3.367	1.043			
11.	I feel exhausted after a day of work and parenting.	1	5	3.367	1.245	−0.402	−0.816
12.	I feel very tired when I wake up in the morning and think about facing the day's tasks.	1	5	3.366	1.248	−0.453	−0.759
13.	I feel that my emotional resources are depleted and I have become numb.	1	5	3.369	1.228	−0.424	−0.766

(a) The constructs (in bold) are the averaged composite scores of their respective items. (b) All items were measured on a 5-point Likert scale (1 = Strongly Disagree, 5 = Strongly Agree). (c) The absolute values of skewness and kurtosis for all items are less than |2| and |7|, respectively, suggesting no severe violation of the univariate normality assumption.

### 3.2 Reliability, common method bias, and measurement model

To assess the internal consistency of the measurement scales, Cronbach's alpha was calculated. The overall scale demonstrated excellent reliability (α = 0.874). All subscales also showed acceptable to good reliability: Digital Parenting Intensity (4 items, α = 0.816), Time Pressure (3 items, α = 0.786), Parenting Guilt (3 items, α = 0.785), and Emotional Exhaustion (3 items, α = 0.792; Table 2).

**Table 2:** Reliability analysis of the measurement scales.

Scale/Dimension	Number of items	Cronbach's alpha
Digital parenting intensity	4	0.816
Time pressure	3	0.786
Parenting guilt	3	0.785
Emotional exhaustion	3	0.792
*Overall questionnaire*	*13*	*0.874*

All Cronbach's alpha values exceed the recommended threshold of 0.70, indicating good internal consistency reliability.

The validity of the factor structure was examined using the Kaiser-Meyer-Olkin (KMO) measure and Bartlett's test of sphericity. The overall KMO value was 0.883, indicating “good” sampling adequacy according to conventional standards (Kaiser, 1974). Bartlett's test of sphericity was significant [χ^2^(78) = 3,509.791, *p* < 0.001], confirming that the correlation matrix was suitable for factor analysis. The KMO values for the individual subscales were all above 0.7, further supporting the factorability of the data (Table 3).

**Table 3:** Kaiser-Meyer-Olkin (KMO) measure and Bartlett's test of sphericity.

Scale/ Dimension	KMO measure	Bartlett's test of sphericity		
		Approx. Chi-Square	*df*	Sig.
Digital parenting intensity	0.804	933.428	6	0
Time pressure	0.704	609.535	3	0
Parenting guilt	0.703	610.018	3	0
Emotional exhaustion	0.707	633.434	3	0
*Overall questionnaire*	*0.883*	*3,509.791*	*78*	*0*

Common method bias was examined using Harman's single-factor test. An unrotated principal component analysis of the 13 measurement items showed that the first factor accounted for 39.77% of the total variance. This value was below the conventional 50% threshold, suggesting that common method bias was not likely to dominate the observed relationships.

We then tested the proposed four-factor measurement model using CFA. The model fitted the data well, χ^2^(59) = 92.13, *p* = 0.004, CFI = 0.990, TLI = 0.987, RMSEA = 0.028, SRMR = 0.026. A single-factor model, in which all items loaded on one general factor, showed poor fit, χ^2^(65) = 1,053.60, *p* < 0.001, CFI = 0.714, TLI = 0.657, RMSEA = 0.146, SRMR = 0.101. These results support treating Digital Parenting Intensity, Time Pressure, Parenting Guilt, and Emotional Exhaustion as four related but distinct constructs.

The standardized factor loadings ranged from 0.678 to 0.771. Composite reliability values ranged from 0.786 to 0.817, and AVE values ranged from 0.529 to 0.560. These values indicate acceptable convergent validity for the four latent constructs.

### 3.3 Correlation analysis

To examine the relationships between the key study variables—Digital Parenting Intensity, Time Pressure, Parenting Guilt, and Emotional Exhaustion—a Pearson correlation analysis was conducted. The results, including means, standard deviations, and inter-correlations, are presented in Table 4.

**Table 4:** Means, standard deviations, and Pearson correlations for study variables (*N* = 713).

Variable	*M*	SD	1	2	3	4
1. Digital parenting intensity	3.185	1.022	-			
2. Time pressure	3.114	1.073	0.459^***^ (0.399, 0.515)	-		
3. Parenting guilt	3.250	1.050	0.375^***^ (0.315, 0.432)	0.472^***^ (0.413, 0.527)	-	
4. Emotional exhaustion	3.367	1.043	0.449^***^ (0.389, 0.505)	0.433^***^ (0.372, 0.490)	0.457^***^ (0.398, 0.512)	-

^***^p < 0.001. Values in parentheses represent the 95% confidence interval for each correlation coefficient.

Pearson correlation analysis was conducted to examine the relationships between the key study variables. As shown in Table 4, all four study variables were positively and significantly correlated with each other at the *p* < 0.001 level. The strength of the correlations, according to Cohen's (1988) conventions, ranged from moderate to large. Specifically, the analysis revealed that: (1) Digital Parenting Intensity showed a significant positive correlation with Emotional Exhaustion [*r* = 0.449, 95% CI (0.389, 0.505), *p* < 0.001]. This indicates that parents who engage more intensely in digital parenting tasks report higher levels of emotional exhaustion. (2) Time Pressure was significantly positively correlated with Emotional Exhaustion [*r* = 0.433, 95% CI (0.372, 0.490), *p* < 0.001]. This suggests that feelings of time pressure are associated with increased emotional exhaustion. (3)Parenting Guilt was also significantly positively correlated with Emotional Exhaustion [*r* = 0.457, 95% CI (0.398, 0.512), *p* < 0.001]. This means that higher levels of guilt related to parenting are associated with higher levels of emotional exhaustion.

Furthermore, the independent variables were also moderately correlated with each other. For instance, the correlation between Time Pressure and Parenting Guilt was *r* = 0.472. And Digital Parenting Intensity was correlated with both Time Pressure (*r* = 0.459) and Parenting Guilt (*r* = 0.375).The presence of these significant, positive bivariate correlations provides initial support for the hypothesized relationships and justifies the subsequent regression and mediation analyses to further investigate these linkages.

### 3.4 Group differences: independent samples *T*-test

To investigate whether there were significant differences in the key study variables based on the parent's gender, an independent samples *t*-test was conducted. The analysis focused on comparing the mean scores of fathers and mothers across the four constructs. The preliminary analysis indicated that only Digital Parenting Intensity showed a statistically significant difference between groups. The results for this significant finding are detailed in Table 5.

**Table 5:** Independent samples *T*-test results for digital parenting intensity by parent gender.

Variable	Parent gender	*N*	Mean	*t*	*p*-value
Digital parenting intensity	Father	386	3.30	3.139	0.002
	Mother	327	3.05		

The degrees of freedom (df) are reported as 657.170 instead of the theoretical 711 because Levene's test for equality of variances was significant (F = 13.218, p < 0.001), indicating unequal variances between groups. Therefore, the corrected 'df' value from the Welch-Satterthwaite approximation was used. CI = Confidence Interval. Cohen's d effect size interpretation: small (d = 0.2), medium (d = 0.5), large (d = 0.8).

As detailed in Table 5, the independent samples *t*-test revealed a statistically significant difference in Digital Parenting Intensity between fathers and mothers, *t* (657.17) = 3.139, *p* = 0.002. The magnitude of the difference, as measured by the mean difference, was 0.250 on the 5-point scale.

The analysis indicates that fathers (*M* = 3.30, SD = 0.93) reported significantly higher levels of Digital Parenting Intensity than mothers (*M* = 3.05, SD = 1.11). To quantify the practical significance of this difference, Cohen's *d* was calculated. The resulting effect size was *d* = 0.259, which is conventionally considered a small to medium effect according to Cohen's (1988) benchmarks. This finding suggests that, within the context of this study, fathers are more intensely engaged in digital parenting activities—such as managing WeChat group tasks, sharing child-related content online, and searching for parenting information—compared to mothers. This counter-intuitive result may reflect evolving parenting roles or specific societal pressures, warranting further investigation in future research. For the other three constructs—Time Pressure, Parenting Guilt, and Emotional Exhaustion—no statistically significant differences were found between fathers and mothers (all *p* > 0.05). This implies that the experiences of time-related stress, feelings of guilt associated with parenting, and overall emotional exhaustion are reported at similar levels by both parent genders in this sample.

### 3.5 Latent-variable structural equation modeling

To test the hypothesized model while accounting for measurement error, we estimated an item-level latent-variable SEM. Digital Parenting Intensity was specified as the predictor, Time Pressure and Parenting Guilt as parallel mediators, and Emotional Exhaustion as the outcome. The model fitted the data well, χ^2^(59) = 92.13, *p* = 0.004, CFI = 0.990, TLI = 0.987, RMSEA = 0.028, SRMR = 0.026, indicating that the proposed model adequately represented the observed covariance structure (Table 6).

**Table 6:** Model fit comparison for confirmatory factor analysis and SEM.

Model	χ^2^	df	CFI	TLI	RMSEA	SRMR
Four-factor CFA/hypothesized SEM	92.13	59	0.990	0.987	0.028	0.026
Single-factor model	1,053.60	65	0.714	0.657	0.146	0.101

CFA, confirmatory factor analysis; SEM, structural equation modeling. The four-factor model specified Digital Parenting Intensity, Time Pressure, Parenting Guilt, and Emotional Exhaustion as distinct latent constructs.

Table 7 presents the standardized path coefficients. Digital Parenting Intensity was positively associated with Time Pressure [β = 0.570, 95% CI (0.483, 0.659)] and Parenting Guilt [β = 0.457, 95% CI (0.365, 0.568)]. In the model including both mediators, Time Pressure was positively associated with Emotional Exhaustion [β = 0.174, 95% CI (0.027, 0.316)], and Parenting Guilt was also positively associated with Emotional Exhaustion [β = 0.342, 95% CI (0.198, 0.465]. The direct path from Digital Parenting Intensity to Emotional Exhaustion remained significant [β = 0.294, 95% CI (0.184, 0.422)], indicating partial mediation.

**Table 7:** Standardized path coefficients for the latent-variable SEM.

Path	β	Boot SE	*z*	*p*	95% CI
Digital Parenting Intensity → Time Pressure	0.570	0.044	12.820	< 0.001	[0.483, 0.659]
Digital Parenting Intensity → Parenting Guilt	0.457	0.052	8.876	< 0.001	[0.365, 0.568]
Time Pressure → Emotional Exhaustion	0.174	0.073	2.379	0.017	[0.027, 0.316]
Parenting Guilt → Emotional Exhaustion	0.342	0.067	5.108	< 0.001	[0.198, 0.465]
Digital Parenting Intensity → Emotional Exhaustion	0.294	0.061	4.799	< 0.001	[0.184, 0.422]

Coefficients are standardized estimates. Bootstrap = 5,000 samples.

### 3.6 Bootstrapped indirect effects and hypothesis testing

The bootstrapped indirect effects are shown in Table 8. The indirect effect through Time Pressure was significant [β = 0.099, 95% CI (0.016, 0.184)], supporting H2. The indirect effect through Parenting Guilt was also significant [β = 0.157, 95% CI (0.090, 0.228)], supporting H3. The total indirect effect was significant [β = 0.256, 95% CI (0.180, 0.340)]. The total effect of Digital Parenting Intensity on Emotional Exhaustion was β = 0.550, 95% CI [0.471, 0.642]. H1 was also supported because the direct path remained significant after both mediators were included.

**Table 8:** Bootstrapped indirect effects in the latent-variable SEM.

Effect	β	Boot SE	*z*	*p*	95% CI
DPI → Time Pressure → Emotional Exhaustion	0.099	0.043	2.329	0.020	[0.016, 0.184]
DPI → Parenting Guilt → Emotional Exhaustion	0.157	0.036	4.401	< 0.001	[0.090, 0.228]
Total indirect effect	0.256	0.041	6.178	< 0.001	[0.180, 0.340]
Direct effect	0.294	0.061	4.799	< 0.001	[0.184, 0.422]
Total effect	0.550	0.044	12.380	< 0.001	[0.471, 0.642]

DPI, digital parenting intensity. Bootstrap = 5,000 samples. Confidence intervals that do not include zero indicate statistically significant effects.

**Figure 1:**
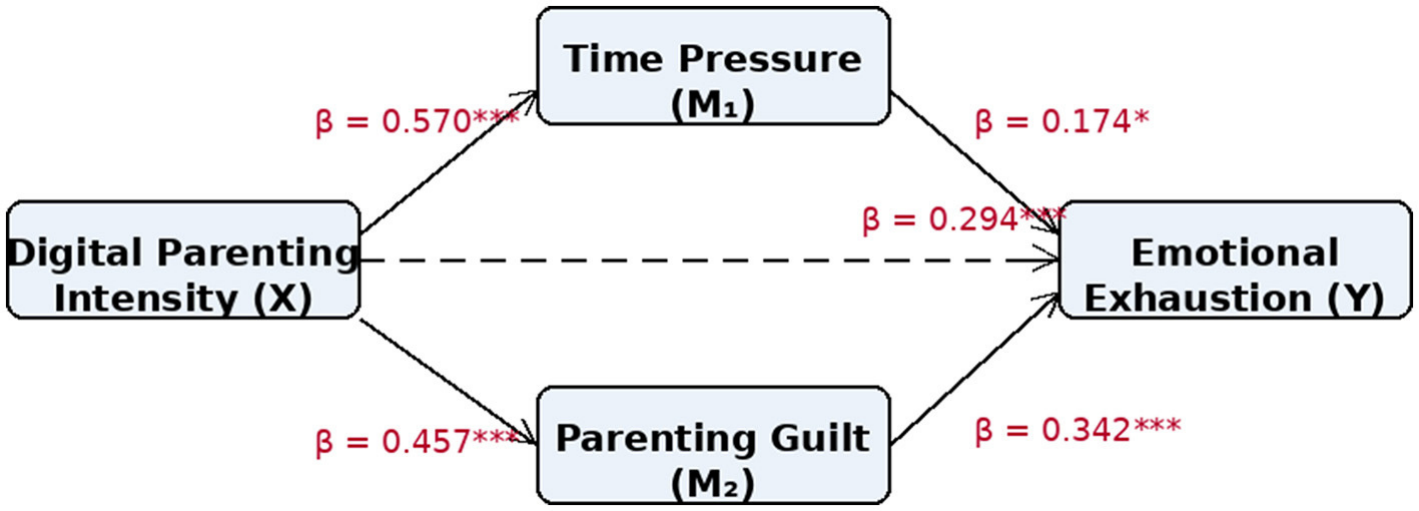
Latent-variable parallel mediation model with standardized path coefficients. ***p < 0.001, *p < 0.05.

Overall, the SEM results supported the proposed model. Digital parenting intensity had a direct association with emotional exhaustion and two significant indirect associations through time pressure and parenting guilt. Because the model was estimated at the item level, these estimates also take measurement error into account (Figure 1).

## 4 Discussion

The findings help clarify what we mean by parenting on a digital leash. In these families, digital tools facilitate communication, but they also keep parents connected to school requests, institutional expectations, peer comparison, and public displays of good parenting. This helps explain the time pressure reported by participants. While work-family conflict is a global issue (Aviad et al., 2024), the form of time scarcity observed here differs from some established accounts. It is not only driven by voluntary long working hours for consumption, as described in some Western contexts (Findling et al., 2024), or by exploitative labor arrangements, as seen in certain labor groups (Çimen and Seçer, 2024). Instead, the pressure appears to come from the simultaneous pull of work commitment and parental companionship. This is the dual ethical squeeze examined in this study. The primary objective was to test how Digital Parenting Intensity is related to Emotional Exhaustion through Time Pressure and Parenting Guilt. The SEM results supported this model.

### 4.1 Key findings

Descriptive statistics indicate that parents in our sample experience moderate to high levels of Digital Parenting Intensity, Time Pressure, Parenting Guilt, and Emotional Exhaustion. These pervasive challenges are not merely individual stressors but reflect a broader cultural dynamic in contemporary China, which we conceptualize as a “dual ethical squeeze” between the work ethic and the companionship ethic.

The latent-variable SEM supported the hypothesized model. Digital Parenting Intensity predicted Emotional Exhaustion, which is consistent with Conservation of Resources (COR) theory. Responding to school notifications, completing online check-ins, searching for parenting information, and managing children's online visibility all require time and attention. When these tasks accumulate, they may reduce parents' cognitive, emotional, and temporal resources.

Beyond the individual stress process, the findings also point to the social context in which this resource loss occurs. We use “dual ethical squeeze” to describe the joint pressure of work dedication and parental companionship. The companionship ethic, promoted through parenting experts, school expectations, and platforms such as WeChat, frames good parenting as requiring regular presence and emotionally engaged time. The work ethic, reinforced by intensive labor cultures, continues to link career commitment with social worth and self-identity (Wu J. J. et al., 2024).

The two mediators show how this squeeze is experienced. First, digital parenting intensity was associated with greater time pressure. Digital tools fragment time and make role boundaries less clear: parents may need to answer work messages while also responding to schools, children, and online parenting communities. Second, digital parenting intensity was associated with parenting guilt. Digital platforms make ideals of good parenting more visible, and parents may interpret their inability to meet these ideals as personal failure. In this sense, the digital leash operates at two levels. It increases practical demands, and it also strengthens internal pressure when parents feel that they are falling short.

### 4.2 The counter-intuitive gender difference

A noteworthy and somewhat counter-intuitive finding emerged from our data: fathers reported significantly higher levels of Digital Parenting Intensity than mothers. This contrasts with traditional narratives that predominantly assign the primary emotional and routine childcare burden to mothers.

One plausible explanation for this result lies in the nature of digital parenting tasks. Activities such as managing WeChat groups, searching for parenting information online, or coordinating extracurricular activities may be perceived as more instrumental and task-oriented—roles that some fathers adopt more readily in the family division of labor (Hiura et al., 2024). However, these interpretations remain speculative and require direct empirical testing. This finding may also reflect an ongoing shift in parenting roles, wherein technology serves as a domain facilitating increased paternal involvement in child-rearing. Given the cross-sectional design, we cannot determine whether fathers' higher scores reflect actual behavioral differences or reporting biases. Future research using time-use diaries or observational methods is needed.

Notably, this does not imply that mothers are less burdened overall. Rather, it suggests that the structural pressures of the “dual ethical squeeze” are increasingly affecting fathers as well. While maternal anxiety may be more visible in online discourse, our data indicate that fathers are also experiencing significant strain. In dual-earner households, fathers are increasingly enmeshed in the demands of the companionship ethic, complicating simplistic narratives of paternal absence (García-Saucedo et al., 2024). This counter-intuitive finding may be understood within China's unique digital parenting ecosystem. In many dual-earner families, mothers are often held responsible for the emotional and relational aspects of childcare, while fathers are expected to handle “instrumental” tasks. Digital parenting activities—such as responding to school WeChat notifications, completing online check-ins, and searching for educational resources—are often framed as administrative or task-oriented duties. These may be delegated to fathers as part of a pragmatic division of labor, especially in families where mothers already bear a heavier load of physical and emotional caregiving. Additionally, the “new ethics of companionship” may pressure fathers to demonstrate their involvement publicly on social media, leading to higher reported intensity. Future qualitative research should explore whether fathers' higher scores reflect actual time spent or a tendency to over-report due to social desirability.

Thus, the core issue extends beyond conventional gendered role divisions. It reflects a broader structural conflict between the work ethic and the companionship ethic, in which digital tools like WeChat intensify parental responsibilities for both genders (Dearden et al., 2013; Rogers and Bales, 2020). These platforms erode boundaries and enable constant surveillance from both professional and domestic spheres, thereby deepening anxiety and time scarcity among all parents (Yin et al., 2024).

Future qualitative research is needed to further unpack the nuances of this finding—particularly to explore whether the nature, context, and emotional impact of digital parenting differ between fathers and mothers, and how these technologically-mediated roles are negotiated within households.

### 4.3 Theoretical and practical implications

Theoretically, this study contributes in three ways. First, it applies COR theory to digital parenting by treating digital parenting tasks as activities that may consume time, attention, and emotional resources. Second, it uses the term “dual ethical squeeze” to capture a context-specific tension between work dedication and intensive companionship in Chinese dual-earner families. Third, the latent-variable SEM treats Digital Parenting Intensity, Time Pressure, Parenting Guilt, and Emotional Exhaustion as distinct constructs and tests the indirect effects while accounting for measurement error. The significant indirect effects through Time Pressure and Parenting Guilt suggest that both external pressure and internalized guilt help explain the link between digital parenting and emotional exhaustion.

*From a practical perspective*, these findings offer valuable insights for parents, educators, and mental health professionals.

*For parents*, it is crucial to recognize the potential psychological costs of digital parenting and to consciously set boundaries. This could involve designating “digital-free” times, batching the processing of digital tasks, and recalibrating expectations to alleviate the burden of perfectionism that fuels guilt.

*For schools and child-related institutions*, it is important to be mindful of the cumulative burden of digital “check-in” tasks assigned to parents. Streamlining communication channels and clarifying the necessity of each task could help reduce the digital load on parents.

### 4.4 Limitations and future research directions

Despite its contributions, this study has several limitations. First, its cross-sectional design prevents definitive causal inferences. While our model is theoretically grounded, longitudinal or experimental studies are needed to confirm the causal relationships proposed. Second, the data relied solely on self-report measures, which may be subject to social desirability and common method bias. Future research could incorporate objective measures of digital device usage or partner-reported data. Third, the sample was drawn from a specific cultural context; the generalizability of the findings to other cultures should be tested. Fourth, although our sample covers multiple cities and sectors, it is over-represented by state-sector employees. Future research should test whether the proposed mediation model holds for dual-earner parents in the private sector, gig economy, or rural areas.

Future research could explore other potential mediators, such as social comparison (e.g., through social media) or partner conflict over digital parenting responsibilities. It could also investigate moderating factors, such as social support or personality traits (e.g., neuroticism), which might buffer or exacerbate the impact of digital parenting on wellbeing.

## 5 Conclusions

This study examined whether digitally mediated parenting is associated with emotional exhaustion among Chinese dual-earner parents. The findings support a clear conclusion: higher digital parenting intensity is associated with higher emotional exhaustion. The latent-variable SEM further shows that this association operates through two parallel pathways: time pressure and parenting guilt.

The results show that digital parenting tasks–including WeChat school check-ins, online parenting information seeking, and public sharing of family life–consume personal resources. These practices can create a digital leash by keeping parents attached to childcare obligations across work, family, and social media contexts. This association was partly explained by time pressure: digital tasks fragmented parents' attention and contributed to a feeling of constant urgency (Li and Wang, 2024). It was also explained by parenting guilt: the always-on digital environment made idealized parenting standards more visible and intensified guilt when parents felt unable to meet them (Robbeets et al., 2024).

A particularly insightful finding was the higher level of digital parenting intensity reported by fathers compared to mothers. This challenges traditional assumptions about the gendered distribution of childcare labor and suggests that technology may be reshaping parental roles in novel ways, possibly by creating new, more instrumentally-focused forms of involvement for fathers.

The implications are both theoretical and practical. The study extends resource-based theories of stress to digital parenting, introduces the dual ethical squeeze as a context-sensitive way to understand strain among Chinese dual-earner parents, and provides latent-variable evidence for time pressure and parenting guilt as parallel mechanisms. For parents, these findings suggest the need to set clearer boundaries around digital parenting tasks. For schools and child-related institutions, they suggest that digital communication should be treated as part of the parental workload rather than as a cost-free channel.

In closing, while digital technology offers undeniable benefits for information and connectivity, its unmanaged use in the parenting realm poses a tangible risk to parental mental health. The path forward does not lie in rejecting technology, but in achieving a sustainable balance—one where digital tools serve as supportive aids rather than oppressive drivers of exhaustion. Future research, particularly of a longitudinal and interventional nature, is essential to further unravel these complex relationships and to develop effective strategies to safeguard the wellbeing of parents in the digital age.

## Data Availability

The raw data supporting the conclusions of this article will be made available by the authors, without undue reservation.

## Appendix A

### Appendix A.1

Table A1 provides the standard interpretation guidelines for the Kaiser–Meyer–Olkin (KMO) measure of sampling adequacy, which is used to assess the suitability of data for factor analysis. The KMO value ranges from 0 to 1, with higher values indicating stronger appropriateness. The table classifies the results into six distinct categories. This classification helps researchers determine whether their dataset is adequate for proceeding with factor analysis.

**Table A1:** Interpretation guidelines for KMO measure.

KMO	Significance
>0.90	Marvelous/Very Good
0.80~0.90	Meritorious/Good
0.70~0.80	Middling/Acceptable
0.60~0.70	Mediocre/Average
0.50~0.60	Miserable/Poor
< 0.50	Unacceptable/Invalid
